# Yoga and Mindfulness as a Tool for Influencing Affectivity, Anxiety, Mental Health, and Stress among Healthcare Workers: Results of a Single-Arm Clinical Trial

**DOI:** 10.3390/jcm9041037

**Published:** 2020-04-07

**Authors:** Giuseppe La Torre, Antonino Raffone, Margherita Peruzzo, Lucia Calabrese, Rosario Andrea Cocchiara, Valeria D’Egidio, Pasquale Fabio Leggieri, Barbara Dorelli, Salvatore Zaffina, Alice Mannocci, YOMIN Collaborative Group

**Affiliations:** 1 Department of Public Health and Infectious Diseases, Sapienza University of Rome, 00185 Rome, Italy; m.peruzzo4@gmail.com (M.P.); rosario.cocchiara@uniroma1.it (R.A.C.); valeria.degidio@uniroma1.it (V.D.); pfabio.leggieri@gmail.com (P.F.L.); barbara.dorelli@uniroma1.it (B.D.); alice.mannocci@uniroma1.it (A.M.); sara.cianfanelli@uniroma1.it (Y.C.G.); 2 Department of Psychology, Sapienza University of Rome, 00185 Rome, Italy; antonino.raffone@uniroma1.it (A.R.); calabrese.lu@gmail.com (L.C.); 3 Occupational Medicine, Bambino Gesù Children’s Hospital, IRCCS, 00165 Rome, Italy; salvatore.zaffina@opbg.net

**Keywords:** yoga, mindfulness, affectivity, anxiety, mental health, stress, healthcare workers, clinical trial

## Abstract

Mindfulness-based interventions have emerged as unique approaches for addressing a range of clinical and subclinical difficulties such as stress, chronic pain, anxiety, or recurrent depression. Moreover, there is strong evidence about the positive effects of yoga practice on stress management and prevention of burnout among healthcare workers. The aim of this study was to conduct a single-arm clinical trial to assess the effectiveness of an intervention based on mindfulness-based stress reduction and yoga in improving healthcare workers’ quality of life. Healthcare workers of two hospitals in Rome were enrolled in a 4-week yoga and mindfulness course. Four questionnaires were administered at different times (Short Form-12 (SF-12), State-Trait Anxiety Inventory (STAI) Y1 and Y2, and Positive and Negative Affect Schedule (PANAS)) to evaluate the efficacy of the intervention. Forty participants took part to the study (83.3 %). The Mental Composite Score-12, that is part of the quality of life assessment, passed from a median of 43.5 preintervention to 48.1 postintervention (*p* = 0.041), and the negative affect passed from a score of 16 in the preintervention to 10 in the postintervention (*p* < 0.001). Both the forms of the STAI questionnaires showed a decrease after the intervention. Yoga and mindfulness administered together seem to be effective to reduce stress and anxiety in healthcare workers, providing them with more consciousness and ability to manage work stressful demands.

## 1. Introduction

In recent years, interest in research investigating mindfulness-based interventions (MBIs) has substantially increased since these interventions have emerged as unique approaches for effectively and comprehensively addressing a range of clinical and subclinical difficulties such as stress, chronic pain, anxiety, or recurrent depression [1,2,3]. Mindfulness can be described as a moment-to-moment awareness that is cultivated by purposefully paying attention to the present experience with a nonjudgmental attitude. It can be implemented through structured programs, including group and individual mental training, and is generally considered to entail the two core components of attention and acceptance [4]. More in details, mindfulness consists of formal meditation exercises (e.g., paying attention to the body, lying on the ground, or walking slowly with a sense of awareness of one’s surroundings) as well as informal exercises (e.g., paying full attention to what one is doing or experiencing at a certain moment) [5]. Moreover, adaptive changes in brain functioning and structure (functional and neural plasticity) related to mindfulness training have been reported in several neuroscientific studies [6,7]. MBIs were initially developed for clinical contexts, but several studies have demonstrated that these interventions improve cognitive performance and emotional reactivity also in nonclinical populations [8,9]. Other studies have shown the effectiveness of MBIs for stress reduction and the enhancement of well-being within the workplace setting [10,11]. Finally, several cross-sectional studies provide indirect evidence that regular mindfulness practice may counteract cognitive ageing, which is indicated by preserved performance in various cognitive tasks as well as preserved neural tissue in older meditators compared to age-matched individuals [12,13].

On the other hand, there is strong evidence, derived from systematic reviews, about the positive effects of yoga practice on stress management and prevention of burnout among healthcare workers [14,15], indicating that it can be incorporated into workplace health promotion in healthcare settings.

There is preliminary evidence that Kundalini Yoga (KY) practice has an immediate effect on salivary cortisol levels as well as on perceived stress after 3 months of practice [16]. Moreover, KY has been used as a treatment for generalized anxiety disorder, and it might convey its effect on symptom severity by reducing somatic symptoms [17]. Moreover, it is useful for improving quality of life and depression symptoms but not for lowering blood pressure [18].

According to the KY philosophy, the primary aim of this discipline is to awake in each individual the full potential of human awareness. This means KY is able to recognize our awareness, to refine it, and to expand that awareness to our unlimited self [16].

The integration of mindfulness and yoga training in our intervention is supported by the evidence that both practices have influences not only on individual brain network, but also on integrated brain states and brain-body interactions [19]. Furthermore, both such time-honored practices can be regarded as synergic in terms of their effects on body and mind states, with particular reference to posture, breath, autonomous nervous system, and the major neural networks involved in the regulation of cognition and emotion [19,20,21].

Further aspects of relevance in our training are the use of a short duration program (4 weeks), which appears not only less costly and with higher odds of attendance among the participants but also easier to be implemented in organizational contexts. In the mostly used MBIs, such as Mindfulness Based Stress Reduction (MBSR), training in formal mindfulness practices is provided, including body scan, sitting meditation, and yoga, with a standard duration of 8 weeks [22] as shown by a recent systematic review [23].

There is some evidence that demonstrates the efficacy of a brief 4-week mindfulness intervention for nurses and nurse aides, with significant improvements in burnout symptoms, relaxation, and life satisfaction [24]. Moreover, another study conducted on veterans with post-traumatic stress disorder demonstrates a beneficial physiological impact on cortisol awakening response with a minimum of 4 weeks of practice [25].

The main purpose of this study, YoMin (Yoga and Mindfulness), was to conduct a single-arm clinical trial (CT) to assess the effectiveness of an intervention based on two types of techniques (MBSR and yoga) in improving mental well-being in healthcare workers (HCWs) of 2 hospitals in Rome.

The hypothesis to be tested was that combined yoga and mindful interventions could be associated with an improvement in the scores concerning quality of life, work-related stress, positive and negative affect, and state and trait anxiety.

## 2. Materials and Methods

### 2.1. Settings and Participants

For this study, healthcare workers of two hospitals in Rome, the teaching hospital “Policlinico Umberto I” and the “Bambino Gesù Children’s Hospital”, were recruited in the period from June 2018 to June 2019. Physicians, nurses, psychologists, and technicians of these hospitals received the intervention in three different editions of the course. Participants who attended at least three of four meetings and who filled out both the entrance and final questionnaires were included in the analysis.

### 2.2. Intervention

The intervention was structured as a 4-week course. Once a week, two 2-h sections in the same day were implemented: one for practicing yoga and one for practicing mindfulness. During the first meeting, an introductory speech about yoga and mindfulness and their effects on stress, burnout, anxiety, and depression was provided to the participants.

Two qualified mindfulness teachers conducted the mindfulness sessions. Each session included four main mindfulness meditation exercises, a period of inquiry about the practice with feedback from the mindfulness teachers, and instructions about homework at the end.

The first mindfulness meditation practice included in each mindfulness session was a “body scan” exercise. It consisted in concentrating and putting the attention on different parts of the body (e.g., toes, back, or head) as well as sensations (such as pain or muscle tension) in the present moment [26]. This was followed by an exercise of mindfulness of the breath sensations or focused attention meditation [26,27]. Then, an exercise of walking meditation was performed in which participants were mindful of their steps during slower walking by sustaining their attention on each step from beginning to the end [28]. Finally, participants performed an open monitoring meditation exercise with mindfulness and open awareness of any arising sensations, feeling, and thought contents in the fields of experience [27]. Participants were instructed to maintain an attitude of emotional acceptance, nonjudgment, and equanimity throughout all the mindfulness activities [22,26,27]. This set and sequence of meditation exercises was chosen to enhance focused and open attention (respectively concentration and mindfulness). They increase individual’s attention and awareness concerning both static and dynamic contents (as their posture or breathing and walking). Finally, they enhance emotion recognition and regulation through emotional acceptance. All these functions have been linked to improvements in physical and psychological well-being as well as cognitive enhancement [4,19,20,27,29,30].

The mindfulness sessions also included periods of inquiry or shared reports from the participants about their mindfulness meditation experiences, with feedback from the mindfulness teachers [22]. Finally, homework instructions were given, including information about maintaining a regular mindfulness meditation practice at home (for at least 20 min per day) as well as to include periods of informal mindfulness practice during everyday life, e.g., performing mindful walking (at normal speed) or being mindful of everyday activities like washing dishes and eating as much as possible. Participants were also instructed to be mindful of their arising emotions, including during working hours, and to observe them mindfully rather than identifying, enacting, or repressing them.

The yoga course was led by a certified professional who delivered the training following many yoga techniques, with specific attention to KY. The aim of this intervention was to provide participants with self-care tools to manage and reduce stress and to be aware of the impact on health and well-being of daily conscious and unconscious activities and functions. Participants were invited to pay specific attention to posture and breathing as a valuable method to manage their minds. During the yoga sessions, the following scheme was followed:

1. 5–10 min of starting session with motivational purposes
2. 30 min of breathing, posture, and sound exercises meant to achieve benefits for work-related stress and burnout (shoulders, neck, hips, and leg muscles were the most treated body district)
3. 20 min of pranayama, aiming at slowing and controlling breathing with diaphragmatic respiration exercises to reduce perceived anxiety. The most used pranayamas were “bastrika” breathing, Nadi Shodana, alternate nostrils breathing, and breath of fire. Participants were asked to do home practice by performing the yoga programs as often as possible, at least twice a week.
4. 20 min of specific meditation exercises with movements and postures for releasing tension, relieving fatigue, and achieving mental well-being.
5. 5 min of mantra (formula repeated many times with meditative purposes)
6. 10 min of relaxation

At the end of the yoga sessions, participants were delivered with brief daily exercises to be executed in home and workplace settings, to manage stress and anxiety, and as a quick solution for obtaining physical and mental relaxation.

### 2.3. Instruments

The main tools used to evaluate the efficacy of the YoMin courses were four questionnaires which were administered to the participants at different times:

1. before the beginning of the course: SF-12, the 15-item form of Karasek, and STAI Y2
2. before starting each 4 h session: STAI Y1 and PANAS
3. at the end of each 4 h session: STAI Y1 and PANAS
4. 21 days after the courses: SF-12, a short form of Karasek, and STAI Y2.

#### 2.3.1. SF-12

This questionnaire was developed as a shorter form of the SF-36 (Short Form Health Survey). SF-36 is a 36-item survey of patient’s health. It was created by the Medical Outcome Study (MOS) [31] and was designed for use in clinical practice and research, health policy evaluations, and general population surveys. Its aim is to furnish a short yet reliable survey directly from the individual and to evaluate his/her health status, assessing eight different health components. The SF-12, an even shorter survey, was later developed and adapted in order to assess a person’s health status [32]. It allows the researcher to investigate mental and physical health through two different summary scores: MCS-12 and PCS-12 respectively.

#### 2.3.2. Job Content Questionnaire

The Job Content Questionnaire (JCQ) was developed by Robert Karasek [33]. It is a self-administered instrument designed to measure social and psychological characteristics of jobs, consisting of 49 questions in its original form. The aim of this questionnaire is to give an evaluation of job demand, control, and support. This is made through different scales:

- decision latitude;
- psychological demands;
- social support;
- physical demands;
- job insecurity.

The most commonly used demand/control model hypothesis predicts that the most adverse reactions of psychological strain occur when the psychological demands are high and the worker’s decision latitude is low: job strain. For this reason, the latter three variables were analyzed in the study. A short form of the original questionnaire (containing 15 questions) was used.

#### 2.3.3. STAI

The State-Trait Anxiety Inventory (STAI) is a psychological inventory consisting of 40 questions on a self-report basis. Charles Spielberger was the main creator of this questionnaire [34]. It was developed to provide both short and reliable scales based on a person’s answers to access two types of anxiety: state anxiety, or anxiety about an event, and trait anxiety, or anxiety level as a personal characteristic. The questionnaire is made of two forms, Y1 and Y2, in order to differently assess the two types of anxiety: state and trait respectively. Each form consists of 20 questions, with a 4-point score per each question, and a range from 20 to 80 per each form. Higher scores correlate with greater anxiety. Two forms of the STAI have been developed: one for children and one for adults. Moreover, not only does it assess anxiety but also it can be used to discriminate between anxiety and depression.

#### 2.3.4. PANAS

The Positive and Negative Affect Schedule (PANAS) is also a self-report questionnaire, consisting in a total of 20-item scale divided in two parts: a 10-item scale for the assessment of positive affect and a homologue for the negative affect. Each item is rated on a 5-point scale of 0 (not at all) to 4 (very much). For instance, positive affect refers to the grade of a person’s positive affectivity regarding emotions, sensation, and sentiments in terms of enthusiasm, energy, self-confidence, and determination. On the other hand, negative affect refers more to negative emotions, such as anger, contempt, guilt, and fear.

The schedule was developed by Watson, Clark, and Tellegen in 1988 in order to provide a better, objective measure of each of these dimensions [35]. Over time, different versions of PANAS have been created to bring modifications and improvements to the first version. It has been mainly used as a research tool in group studies, but it has the potential to be also used in the clinical practice [36].

### 2.4. Statistical Analysis

The statistical analysis included descriptive statistics and univariate analysis. For continuous variables (e.g., age), mean ± standard deviations or median and range (minimum and maximum) were used, whereas categorical data were presented as numbers and percentages.

The primary outcome was considered the quality of life, while the secondary outcomes were represented by decision latitude, job demand and job strain, positive and negative affect, and state and trait anxiety.

Sample size was then calculated on the hypothesis of an increase of 20 % of the MCS. A previous study of our team [37] showed an MCS score of 40.48 (SD 10.56), and in order to reach a 20 % increase with a power of 80 % and a sensitivity of 95 %, at least 24 healthcare professionals should have been recruited.

To evaluate differences between pre- and postintervention results, the univariate analysis of scores was applied using the Wilcoxon test.

Moreover, *t*-test and Mann-Whitney test were used to evaluate differences between groups for the delta scores.

Finally, a multiple linear regression analysis was performed having as dependent variables the delta scores and as independent variables the following: age (continuous variable); gender (males as the reference group); healthcare professional type (physicians vs other HCWs); and type of hospital (teaching hospital vs pediatric hospital). The goodness of fit of the models was assessed using R^2^.

All statistical analyses were performed using the Statistical software SPSS version 25.0 for Windows (IBM SPSS Statistics 25). The level of statistical significance was set at *p* < 0.05.

## 3. Results

**Figure 1:**
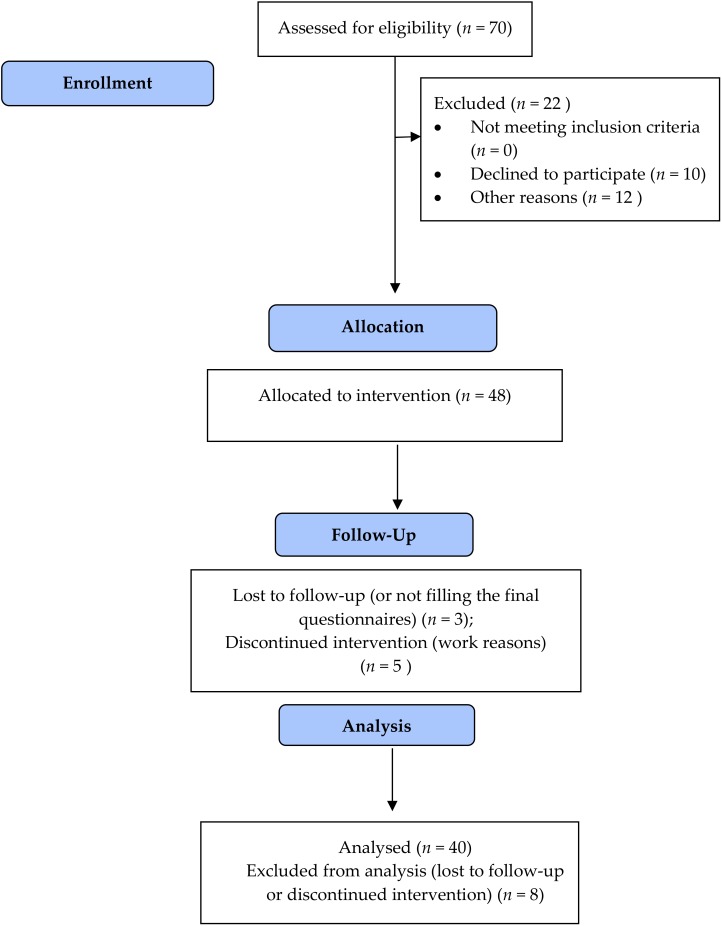
Flow Diagram of the trial.

Out of the 48 enrolled HWCs, 36 women and six men underwent the study (83.3 %) (Figure 1). The dropouts were mainly due to work reasons or not filling the final questionnaires. For this reason, data from these HCWs were excluded from the analysis.

Twenty-eight were professionals of Policlinico (Teaching hospital), and 12 came from OPBG (Paediatric hospital). The mean age was 47.3 years. They were divided per profession into three main groups, resulting in 13 physicians, 15 nurses, and 12 other HCPs (such as biologists, lab technicians, and psychologists). Table 1 shows more details about the participants’ characteristics.

### 3.1. Univariate Analysis: Comparison Pre- and Postintervention

The scores of the variables (the different questionnaires) were analyzed with the Wilcoxon test in order to assess existing differences between pre- and postintervention values. The median was considered. Table 2 describes the results.

#### 3.1.1. SF-12

The two considered scales that assessed the global quality of life (MCS-12 and PCS-12) both showed interesting results. The MCS-12 score passed from a preintervention median of 43.5 to a postintervention median of 48.1 (*p* = 0.041), demonstrating that a visible improvement in the mental health of the participants was obtained. Also, the PCS-12 showed some improvements, though not statistically significant (*p* = 0.09), with scores of 49.4 in the preintervention and of 54.8 in the postintervention assessment.

#### 3.1.2. JCQ

The analysis of this questionnaire led to three scores: decision latitude, job demand, and job strain. As results, the first score showed no difference between pre- and postintervention, with a median of 54 in both cases (*p* = 0.419). The job demand score passed from 27 to 26 (*p* = 0.373), suggesting a slight but not statistically significant improvement. Finally, the job strain score improved, passing from 0.76 to 0.72 (*p* = 0.100), but this change was not statistically significant.

#### 3.1.3. PANAS

The analysis of this questionnaire and the following results were separated: the positive affect passed from 37 preintervention to 36.5 postintervention (*p* = 0.762), showing no significant improvement; on the other hand, the negative affect passed from a score of 16 in the preintervention to 10 in the postintervention (*p* < 0.001), showing a consistent gain.

#### 3.1.4. STAI

Both the forms of the questionnaires showed a decrease in the scores after the intervention. In particular, the Y1 form (state anxiety) changed from 62 in the preintervention to 45.5 in the postintervention (*p* < 0.001), showing a clear reduction of the anxiety level. Similar results were obtained with the Y2 form (trait anxiety), for which the score decreased from 46 in the preintervention to 43.5 in the postintervention (*p* = 0.009).

### 3.2. Multivariate Analysis: Linear Regression on Delta Scores

A further analysis was conducted in order to see whether some characteristics of the participants could reveal any association on the dependent variables (pre- and postintervention differences in the scores) due to the intervention. The results showed almost no difference among all the analyzed variables. As we can see in Table 3, as for the SF-12, neither MCS-12 nor PCS-12 revealed any impact of all the variables considered. Interestingly, the JCQ showed some differences in the results for the type of the hospital. In particular, the delta score for the decision latitude showed a standardized beta value of −0.422 (*p* = 0.02, R^2^ = 0.171), meaning that the HCWs of OPBG benefited more from the intervention compared to the HCWs of the teaching hospital. Consequently, job strain also resulted in being affected by the type of hospital, with a beta value of the delta score of 0.455 (*p* = 0.015, R^2^ = 0.189), yet it must be considered that the Karasek itself did not report evident effects of the intervention. PANAS showed no influence of all the variables considered. As far as concerns the STAI, the Y1 form (state anxiety) revealed no difference for all the variables; conversely, the Y2 form (trait anxiety) for the type of HCW had a beta coefficient of the delta score of −0.326 (*p* = 0.05, R^2^ = 0.130), revealing that the workers who benefited the most from the intervention were the physicians.

## 4. Discussion

The healthcare environment can be a big source of stress for several factors. Working in a hospital means facing everyday with administrative issues and difficult cases, either for diagnosis or management of some pathologies, and the permanent possibility of an emergency. Other supplemental stressors often play a major role in the worsening of work-related stress (WRS). A bad work environment, with unpleasant colleagues or supervisors, lack of transparency and honesty among employees, bad conditions of the workplace (i.e., lack of adequate instruments), and most of all the persistent contact with the patients which means people suffering, can emotionally disrupt HCWs in addition to a physical strain, leading them to perceive high levels of stress.

The main purpose of this study was to provide HCWs with a tool to handle daily work stressors, to therefore manage anxiety and depression, and to overall obtain an improvement in their quality of life. Both the interventions purposed, yoga and mindfulness, have already showed to positively affect different aspects of mental and physical health.

Fang and Li’s study demonstrated the effects of Yoga focusing on sleep problems in nurses [38]. Riley’s work, apart from estimating how yoga can improve the physical and psychological health status of staff, made a comparison between the effectiveness of yoga programs and cognitive training programs in determining a better mental well-being and a reduction of stress-related consequences, with yoga showing better results [39]. The yoga program discussed in the study of Klatt et al. emphasizes the advantages of an activity that prove to be feasible and adaptable to the working environment [40]. Also, for mindfulness, many studies revealed the effectiveness of this tool for keeping WRS under control. Lamothe et al., for example, in their review summarized the results of many studies, stating that MBSR interventions decrease the level of perceived stress, burnout, and anxiety [41]. Spinelli and Smith also did in their reviews. [42,43]. Stillwell considered pre- and postintervention effects in healthcare students in order to assess the immediate effects of MBSR techniques and other MBIs [44]. Again, the results were positive, with a decrease in perceived stress and anxiety.

In the present study, differently from the other considered studies, the two interventions (yoga and MBSR) were administered together, so the discussion of the results can only partially be referred to the single intervention.

First of all, according to the results, a further confirmation of the effectiveness of yoga and mindfulness can be drawn, since almost all the measured parameters showed an improvement. The most evident outcome was the reduction of anxiety. For instance, a major decrease resulted from the Y1 form of STAI, which passed from a score of 62 before the session to 45.5 at the end of the session, meaning that the most benefit for HCWs was an immediate decrease of anxiety level. More interestingly, also the Y2 from of STAI (which measured the trait anxiety) showed positive significant results, passing from 46 before the beginning of the course to 43.5 at 3–4 weeks after the end of the course. If the first outcome confirms that both yoga and mindfulness are helpful in decreasing anxiety about events, as it is intuitive, the second outcome states that the same interventions also have an impact on the anxiety level as a personal characteristic, having therefore a long-time effect. However, more evaluations over time should be done to see for how much time the effect lasts. O’Driscoll analyzed studies that considered follow-up at 6 months after the interventions, but no sustained results were found [45]. This could mean that a continuous practice of yoga and mindfulness courses may be useful to maintain the obtained results. The other questionnaire administered during the course, PANAS, also showed some interesting evidences. In particular, the positive affect had no significant difference in pre- and postintervention scores, meaning that aspects such as energy, enthusiasm, and self-confidence were not affected, but the negative affect had an improvement, with a decrease of scores from 16 preintervention to 10 postintervention. This means that aspects such as fear, weakness, and anger were effectively diminished. These results can be considered in line with the STAI ones, since both the questionnaires investigate similar aspects of the individual’s psychological traits. Furthermore, an improvement in these characteristics is directly linked with WRS, since lower levels of anger, sense of weakness, and fear clearly help HCWs to handle the stressful situations that can occur in the workplace. As for SF-12 outcomes, the most relevant results were the ones about the HCWs’ mental health. An increase in the SCM-12 score from 43.5 to 48.1 stands for a visible improvement of the HCWs’ mental well-being, which in turns means a decrease of perceived stress. On the other hand, no results were found regarding physical health. A prolonged course of Yoga may be useful under this aspect. Lastly, the JCQ, which was the main work-addressed questionnaire, showed no change in both perceived decision latitude, job demand, and job strain. This is understandable, since the type of intervention was aimed to focus on the individual rather than on the group. Moreover, the outcomes measured by this questionnaire are somehow subordinated to one’s perceived stress and anxiety, so even though no direct effects have been showed, it does not mean that the aim of the intervention was not achieved. Rather, it confirms that yoga and mindfulness have a global effect on the individual’s mental well-being regardless of the workplace conditions yet represent a good instrument to reduce WRS.

Interesting considerations about the multivariate analysis need to be made. The most relevant finding is related to the STAI Y2 form (trait) that considered the type of profession. For instance, a major decrease in the score was reached for the physicians. This means that the intervention resulted more usefully in decreasing anxiety in physicians rather than in the other HCWs. The other main difference was found in the JCQ results considering the type of hospital. In fact, even though no relevant overall results were found for the univariate analysis, a comparison between HCWs in teaching hospitals and those in pediatric hospitals revealed that a better effect was reached for the second ones, with decision latitude and job strain levels being lower in these. As for the other multivariate analysis, no significant differences were evidenced. In fact, this is a positive result because it means that the intervention had basically the same effects on all kinds of participants, hence representing a tool to handle WRS for all HCWs.

Our idea is that this tool represents a collective protection provision to be used for those activities in which risk assessment demonstrates the presence of work-related stress, and this actually represents the second innovative aspect linked to this intervention.

Moreover, the European Agency for Safety and Health at Work carried out quantitative analyses on data from the second European Survey of Enterprises on New and Emerging Risks (ESENER-2) focusing on psychosocial risks [46]. In this report, it can be found the evaluation on synergistic role of occupational physician and psychologist in the management of psychosocial risks. In particular, this survey highlighted the lack (16 %) of psychologists inserted into the departments of occupational medicine in many European countries. In fact, this integration allows the implementation of health prevention activities such as that reported in this study and highlights how the combined action of the clinical and risk assessment skills of the occupational physician associated with the psychologist’s health activities can produce good results as in this study. The holistic approach of our occupational medicine departments represented the skills necessary for the physical and psychological well-being of health professionals.

### 4.1. Limitations

Despite the encouraging results, some limitations regarding this study need to be acknowledged. A major problem was represented by the setting of the study, since at the beginning of the course, more than 70 participants applied, filling out the preintervention questionnaires, but then, some of them could not complete the course, mainly due to work shifts. Some other participants did not complete the final questionnaires, thus being excluded from the study. This reduction of the sample size unavoidably affected the reliability of the study, which is anyway significant. Another main weak point was the single-arm design which has no control arm and consequently the lack of a control group, which would have allowed to better highlight the measured outcomes. The absence of follow-up also represented a limitation for this study. The reasons were similar to the ones for the reduction of the sample size. This was a main problem in most of the analyzed studies, since the researches seldom could follow the participants at different months after the intervention. Furthermore, in our case the course was quite short, lasting only 4 weeks. A prolonged course could have given more reliable findings regard the maintenance of the achieved effects as well as a follow-up at 6 months or 1 year to record any long-lasting effect.

### 4.2. Directions for Future Research

Even though the literature is rich in studies about the use of yoga and mindfulness as tools to handle WRS in HCWs, most of them reported different limitations that were shared by this study. Hence, since it is likeably that these instruments are valuable, some efforts to overcome these limitations need to be made in order to better assess their use. Firstly, a prolonged follow-up should be made, as said before, to evaluate whether these interventions have some long-lasting effects in reducing HCWs’ WRS. Another suggestion could be an integration in the evaluation of the outcomes. Physical parameters measurements, such as blood pressure, or biochemical ones, such as cortisol levels, would be strong instruments to reinforce the evidence. Finally, more attention to adherence of the participants should be accounted in order to obtain a larger sample size and to avoid any possible selection bias.

## 5. Conclusions

The hospital environment can be a big source of stress for healthcare workers, leading to different consequences on their quality of life, both on HCWs’ mental and physical health and on the healthcare system itself. The aim of this study was to suggest a method to handle the difficult environment in which HCWs work. Results from this study suggest that yoga and mindfulness seem to be effective in reducing stress and anxiety in HCWs, providing them with more consciousness and ability to manage work stressful demands. It has an even greater meaning if we consider that, only in the last years, more attention has been paid on HCWs’ mental wellness, and burnout has been recently recognized as a real syndrome. Moreover, not only could these effects be positive for the work life but also they could apply out of the work setting, since both these interventions aim to give to the individual the possibility to concentrate on himself and thus the ability to master the surrounding life events. In conclusion, more interest and participation should be given to this matter by hospitals, since it is becoming more and more important to take care of the healthcare workers’ well-being.

## Figures and Tables

**Table 1:** Participants’ characteristics.

Variable	*n* (%) or Mean (SD)
Gender	
Females	34 (85)
Males	6 (15)
Age	47.3 (10.9)
Health Professions	
Physicians	13 (32.5)
Nurses	15 (37.5)
Other HCW	12 (30)
Hospital	
Teaching hospital	28 (70)
Pediatric hospital	12 (30)

HCW—Healthcare workers.

**Table 2:** Univariate analysis for the variables of interest: comparison pre-and post-course.

Variables	Pre-Course Median (Min–Max)	Post-Course Median (Min–Max)	*p*-Value
SF-12:			
MCS-12	43.5 (21.5–59.4)	48.1 (20.4–56.7)	**0.041**
PCS-12	49.4 (36.2–65.5)	54.8 (36.6–60.5)	0.09
JCQ:			
Decision latitude	54 (42–66)	54 (4– 68)	0.419
Job demand	27 (11–41)	26 (12–38)	0.373
Job strain	0.76 (0.28–1.18)	0.72 (0.36–1.08)	0.100
PANAS:			
Positive	37 (21–49)	36.5 (18–50)	0.762
Negative	16 (10–34)	10 (8–26)	**<0.001**
STAI Y2 (trait)	46.0 (38–56)	43.5 (32–55)	**0.009**
STAI Y1 (state)	62 (55–72)	45.5 (34–52)	**<0.001**

SF-12—Short Form-12; MCS—Mental Composite Score; PCS—Physical Composite Score; JCQ—Job Content Questionnaire; PANAS—Positive and Negative Affect Schedule; STAI—State-Trait Anxiety Inventory.

**Table 3:** Multivariate analysis—linear regression analysis on the delta (pre-and postintervention difference) scores.

Variables	Gender	Age	Physicians	Teaching Hospital	R^2^
SF-12					
MCS-12	−0.005 (0.979)	0.056 (0.772)	0.089 (0.606)	−0.075 (0.702)	0.014
PCS-12	−0.05 (0.765)	−0.205 (0.268)	0.123 (0.457)	0.373 (0.052)	0.111
Karasek					
Decision latitude	−0.074 (0.646)	0.092 (0.604)	0.050 (0-753)	−0.422 (0.024)	0.171
Job demand	−0.058 (0.728)	−0.138 (0.458)	−0.176 (0.296)	0.243 (0.204)	0.086
Job strain	−0.032 (0.838)	−0.204 (0.248)	−0.141 (0.372)	0.455 (0.015)	0.189
PANAS					
Positive	0.0 (0.999)	0.284 (0.127)	0.227 (0.173)	−0.075 (0.689)	0.111
Negative	−0.185 (0.276)	0.010 (0.959)	0.098 (0.559)	−0.171 (0.370)	0.082
STAI Y2 trait	0.115 (0.483)	−0.128 (0.481)	−0.326 (0.05)	−0.122 (0.510)	0.130
STAI Y1 state	0.089 (0.601)	0.197 (0.298)	0.053 (0.755)	0.082 (0.671)	0.061
